# Risk factors for chronic periodontitis in Sri Lankan adults: a population based case–control study

**DOI:** 10.1186/s13104-017-2778-3

**Published:** 2017-09-07

**Authors:** Nimali Wellapuli, Lilani Ekanayake

**Affiliations:** 1 Ministry of Health, Nutrition and Indigenous Medicine, Colombo, Sri Lanka; 2 Faculty of Dental Sciences, University of Peradeniya, Peradeniya, Sri Lanka

**Keywords:** Case–control study, Chronic periodontitis, Risk factors, Sri Lanka

## Abstract

**Objective:**

To determine risk factors for chronic periodontitis in 30–60 year olds in Sri Lanka. Cases and controls for this population based unmatched case–control study were identified from a broader cross-sectional study which was conducted to determine the prevalence of chronic periodontitis in 30–60 year old adults in Colombo district Sri Lanka. The study included 694 cases and 706 controls. Data were collected by means of a pre-tested interviewer administered questionnaire to obtain information about socio-demographic and behavioural factors, a physical examination to record anthropometric measurements and an oral examination.

**Results:**

Being a male, a Muslim, belonging to the 45–60 year old age group, having less than 12 years of education, using the finger to clean teeth, current smoking, current betel quid chewing, self-reported diabetes and hypertension emerged as risk factors for chronic periodontitis.

**Conclusions:**

Several socio-demographic and behavioural factors as well as co-morbid conditions emerged as independent risk factors for chronic periodontits in this population. The findings could be used for planning programmes to reduce the burden of chronic periodontits in Colombo district Sri Lanka.

## Introduction

Chronic periodontitis is an inflammatory disease that affects 11% of the global population [1]. Further it has a significant impact on the quality of life of individuals [2]. Studies from many developed countries indicate that several modifiable and non-modifiable risk factors such as socio-economic position, smoking, alcohol, diabetes, obesity, hypertension, stress and genetic factors are associated with the disease [3, 4].

As chronic periodontits results from a complex interaction between bacterial infection, host response and risk factors [5], it is possible that population specific characteristics such as ethnicity, genetic, behavioural and environmental factors may operate in different ways in the expression of periodontitis in different populations. Therefore risk factors for chronic periodontis for populations from developed countries may not necessarily be the same for populations from developing countries.

The prevalence of periodontal disease is high (90%) in Sri Lanka [6] but information on risk factors for periodontitis is lacking for Sri Lankans. The available information on this topic is limited to the associations between tobacco use, oral hygiene practices and periodontal disease [7]. Therefore there is a need to identify what additional factors are associated with chronic periodontitis in Sri Lankans. The aim of the present study was to determine risk factors for chronic periodontitis in 30–60 year olds in Sri Lanka.

## Methods

Cases and controls for this population based unmatched case–control study were identified from a broader study that was conducted to determine the prevalence of chronic periodontitis in 30–60 year old adults in Colombo district, Sri Lanka [8]. The sample of that study included 1400 participants who have been residents of the district for a continued period of 1 year or more. Pregnant women, temporary lodgers and the physically and mentally challenged were excluded.

Case definitions developed by the Centers for Disease Control and Prevention/American Academy of Periodontology (CDC/AAP) were used to define moderate and severe periodontitis [9]. Severe periodontitis was defined as having ≥2 inter-proximal sites with clinical attachment loss (CAL) of ≥6 mm (not on the same tooth) and ≥1 inter-proximal sites with probing depth (PD) of ≥5 mm (not on the same tooth) and moderate periodontitis was defined as ≥2 inter-proximal sites with CAL of ≥4 mm (not on the same tooth) or ≥2 inter-proximal sites with PD of ≥5 mm (not on the same tooth). A participant with either moderate or severe periodontitis was considered as a case.

OpenEpi sample size calculator for unmatched case–control studies was used to calculate the sample size [10]. Overweight/obesity is considered as a risk factor for chronic periodontitis [11]. Therefore the prevalence of overweight/obesity (49%) in 20–60 year olds in Colombo district was considered to calculate the sample size [12]. Assuming that the exposure rate among controls is 49% and to detect an odds ratio of 2 with a power of 80%, at a significance level of 5%, a minimum of 137 cases and controls were needed. However based on the CDC/AAP definition, 694 cases with moderate/severe periodontitis and 706 controls were identified from the main study [8]. Therefore to enhance the power of the study, all 694 cases and 706 controls identified from that study were included in the present study. A detailed description of the methodology of the cross-sectional study is described elsewhere [2].

A pre-tested interviewer administered questionnaire was used to obtain information on socio-demographics, behavioural factors and co-morbid conditions. A physical examination was carried out to record anthropometric measurements. The oral examination included the assessment of probing pocket depth (PPD) and clinical attachment loss (CAL) on six surfaces of all teeth (mesio-buccal, buccal, disto-buccal, disto-lingual, lingual and mesio-lingual surfaces) excluding the third molars. All periodontal parameters were recorded using the University of North Carolina (UNC)—12 probe (Hu-Friedy Manufacturing Co, Chicago, USA). Anthropometric measurements included standing body height, weight and waist circumference. Measurements were made when the participant was in upright position, wearing light indoor clothing without footwear or any heavy items in the pockets. Data collection took place at the participant’s home. The first author (calibrated against a professor in Periodontology) recorded the periodontal parameters under an artificial head light when the participant was seated on a chair. Two field assistants, one of whom was conversant in both the Sinhala and Tamil languages administered the questionnaire to the participants. Anthropometric measurements were recorded by a trained field assistant. In order to determine intra-examiner variability of periodontal parameters 5% of the sample was re-examined. Kappa statistics of PPD and CAL measurements were 0.87 and 0.92 respectively.

Data were analysed using SPSS 20.0 software (SPSS Inc, Chicago, Il, USA). Body mass index (BMI) cut-offs were defined based on the proposed WHO categories for Asians: underweight (<18.5), normal weight (18.5–23.0), overweight (>23.0 to <27.5) and obese (≥27.5) [13]. Smoking status was defined as; never if never smoked during life time; former if had discontinued for more than 1 year before data collection and current if smoked either daily or on some days at the time of data collection. Lifetime exposure to smoking was determined in terms of pack-years (number smoked per day/20 × number of years of smoking) for both former and current smokers. Chi square test was used to determine the differences in categorical exposure variables in cases and controls while Mann–Whitney test was used to assess the difference in pack-years smoked. Multiple backward stepwise conditional logistic regression analysis was used to determine the independent effects of exposure variables on chronic periodontitis. A level of p <0.05 significance was considered as the cut-off to retain an exposure variable in the adjusted model. Only those variables that were associated with chronic periodontitis at p <0.05 level in the unadjusted logistic regression models were included in the final model. Smoking in pack-years was included as a continuous variable. Interactions between alcohol × smoking, smoking × alcohol × betel chewing on chronic periodontitis were also assessed.

## Results

The mean age of cases and controls were 47.1 (SD 9.5) and 40.1 (SD 9.1) years respectively. Sixty two percent of the cases and 41% of the controls were males.

Cases and controls differed significantly in relation to sex, age group, ethnicity, level of education and current occupation category (Table 1).

**Table 1:** Comparison socio-demographic exposure variables between cases and controls

Variable	Cases (694)		Controls (706)		p value
	n	%	n	%	
Sex					
Female	261	37.6	419	59.3	<0.001
Male	433	62.4	287	40.7	
Age group (years)					
30–44	298	42.9	374	53.0	<0.001
45–60	396	57.1	332	47.0	
Ethnicity					
Sinhala	545	82.4	581	79.2	0.033
Tamil	75	11.5	81	10.9	
Muslim	68	6.1	43	9.9	
Level of education (years)					
0–5	86	12.4	50	7.1	<0.001
6–10	277	39.9	197	27.9	
11–12	299	43.1	401	56.8	
>12	32	4.6	58	8.2	
Monthly household income (Rupees)					
≤20,000	494	71.2	512	72.5	0.57
>20,000	200	28.8	194	27.5	
Current occupation^a^					
Low category	568	81.8	526	74.5	0.001
High category	126	18.2	180	25.5	

For all variables but ethnicity, the total number of cases and controls were 694 and 706 respectively. With regards to ethnicity, seven participants who belonged to minor races were excluded in the analysis; cases = 688 and control = 705)

^a^Low = unskilled/skilled larbourer, unemployed for males, housewife, lower business; high = professional, managerial, clerical, technical, upper business

Except tooth brushing frequency, all behavioural variables considered differed significantly between cases and controls. Self-reported diabetes and self-reported hypertension were significantly higher in cases than in controls (Table 2).

**Table 2:** Comparison of behavioural exposure variables and co-morbid conditions between cases and controls

Variable	Cases (694)		Controls (706)		p value
	n	%	n	%	
Mode of tooth cleaning					
Brush	673	97.0	703	99.6	<0.001
Finger	21	3.0	3	0.4	
Material used for tooth cleaning					
Toothpaste	666	96.0	698	98.9	0.001
Other	28	4.0	8	1.1	
Tooth cleaning frequency					
Once/day	125	18.0	105	14.9	0.11
>Once/day	569	82.0	601	85.1	
Smoking					
Never	432	62.2	603	85.4	<0.001
Former	80	11.5	45	6.4	
Current	182	26.2	58	8.2	
Lifetime exposure to smoking in pack-years^a^	0	(0–45)	0	(0–79.2)	<0.001
Betel quid chewing					
No	583	84.0	666	94.3	<0.001
Current chewer	111	16.0	40	5.7	
Alcohol consumption					
Never	299	43.1	442	62.6	<0.001
Ever	395	56.9	264	37.4	
Self-reported diabetes					
No	590	85.0	658	93.2	<0.001
Yes	104	15.0	48	6.8	
Self-reported hypertension					
No	562	81.0	647	91.6	<0.001
Yes	132	19.0	59	8.4	
Overweight/obese^b^					
No	292	42.1	266	37.7	0.09
Yes	402	57.9	440	62.3	
Abdominal obesity^c^					
Normal	318	45.8	325	46.0	0.93
With abdominal obesity	376	54.2	381	54.0	

^a^Median, (minimum and maximum) values given

^b^Overweight/obese based on BMI > 23.0 cut-off for Asian populations

^c^Abdominal obesity based on waist circumference ≥80 cm and ≥90 cm for adult females and males respectively for Asian populations

Accordingly to the multiple backward stepwise logistic regression analysis being a male, a Muslim, belonging to the 45–60 year old age group, having 0–5, 6–10, and 11–12 years of education compared to >12 years of education, using the finger to clean teeth, current smoking, current betel quid chewing, self-reported diabetes and self-reported hypertension were independently associated with chronic periodontitis. None of the interactions considered was significant in the final model (Table 3).

**Table 3:** Factors associated with chronic periodontitis (backward stepwise multiple logistic regression analysis)

Variable	Unadjusted		p value	Adjusted		p value
	OR	95% CI		OR	95% CI	
Sex						
Female	1.00			1.00		
Male	2.42	1.95–3.00	<0.001	1.97	1.47–2.65	<0.001
Age group (years)						
30–44	1.00			1.00		
45–60	4.29	3.41–5.40	<0.001	4.19	3.23–5.44	<0.001
Ethnicity						
Sinhala	1.00			1.00		
Tamil	0.99	0.71–1.38	0.94	0.77	0.51–1.14	0.19
Muslim	1.69	1.31–2.51	0.01	1.63	1.03–2.58	0.04
Level of education (years)						
>12	1.00			1.00		
11–12	1.35	0.86–2.13	0.20	1.68	1.00–2.83	0.05
6–10	2.55	1.60–4.07	<0.001	2.80	1.63–4.81	<0.001
0–5	3.12	1.79–5.43	<0.001	2.39	1.23–4.61	0.01
Monthly household income (Rupees)						
≤20,000	1.00					
>20,000	1.07	0.85–1.35	0.58			
Current occupation						
Low category	1.00					
High category	0.65	0.50–0.84	0.001			
Mode of tooth cleaning						
Brush	1.00			1.00		
Finger	7.31	2.17–24.63	0.001	4.30	1.05–17.61	0.04
Material used for tooth cleaning						
Toothpaste	1.00					
Others	3.67	1.66–8.11	0.001			
Tooth cleaning frequency						
Once/day	1.00					
>once/day	0.80	0.60–1.06	0.11			
Smoking						
Never	1.00			1.00		
Former	2.48	1.69–3.65	<0.001	1.21	0.76–1.93	0.43
Current	4.38	3.18–6.03	<0.001	3.27	2.21–4.85	<0.001
Smoking in pack-years	1.15	1.10–1.20	<0.001			
Betel quid chewing						
No	1.00			1.00		
Current chewer	3.17	2.17–4.63	<0.001	2.05	1.34–3.14	0.001
Alcohol use						
Never	1.00					
Ever	2.21	1.79–2.74	<0.001			
Alcohol × smoking						
Never/never	1.00					
Ever/former smoker	2.37	1.61–3.50	<0.001			
Ever/current smoker	4.02	2.89–5.60	<0.001			
Alcohol × betel chewing × smoking						
Never/never/never	1.00					
Ever alcohol user × current chewer × former smoker	3.43	1.36–8.64	0.009			
Ever alcohol user × current chewer × current smoker	4.45	2.13–9.30	<0.001			
Self-reported diabetes						
No	1.00			1.00		
Yes	2.15	1.69–3.46	<0.001	1.55	1.01–2.36	0.04
Self-reported hypertension						
No	1.00			1.00		
Yes	2.58	1.86–3.57	<0.001	1.77	1.20–2.60	0.004
Overweight/obese						
No	1.00					
Yes	0.83	0.67–1.03	0.09			
Abdominal obesity						
No	1.00					
Yes	1.01	0.82–1.25	0.94			

## Discussion

This study is the first to identify a range of risk factors for chronic periodontitis in a large sample of Sri Lankan adults using CDC/AAP case definitions.

Age was the strongest socio-demographic risk factor for chronic periodontitis, a finding consistent with other studies [7, 14]. It is well established that ageing per se does not increase the susceptibility to chronic periodontitis but greater periodontal destruction observed with increasing age is due to the cumulative effect of previous disease activity [15]. However age-dependent alterations in innate immunity and inflammatory status could increase the susceptibility to periodontitis [16]. Chronic periodontitis is more prevalent in men than in women [17, 18] and the sex difference has been attributed to life styles differences [3]. But sex emerged as an independent risk factor following adjustment for confounding effects of life style factors such as tooth cleaning habits, smoking and betel quid chewing. Sex differences in periodontal disease may also be due to gender-based heterogeneity in immune responses [19]. Being a Muslim was a risk factor for chronic periodontitis and the first study to show an association between ethnicity and chronic periodontitis among Sri Lankans. The link between race/ethnicity and periodontitis may be due an indirect effect through mediators such as education, income and occupation rather than to confounders [20]. Socio-economic position whether assessed in terms of education, occupation or income is a predictor of chronic periodontitis but education is more important than income or occupation [21]. Only low educational attainment emerged as a risk factor for chronic periodontitis indicating that different socio-economic indicators contribute differently to chronic periodontitis in different populations.

Those using the finger to clean their teeth were more likely to be at risk of chronic periodontitis than those who used the toothbrush. Use of finger will not remove dental plaque which is the main aetiological agent for chronic periodontitis. A similar finding has been reported previously [7].

Consistent with previous studies [22], current smoking emerged as a strong risk factor for chronic periodontitis but former smoking was not. Smoke cessation has a positive influence on the occurrence of periodontitis [23]. However according to some studies both current and former smokers are at a higher risk of chronic periodontitis than non-smokers [24]. A dose–response association between smoking and periodontitis has been observed [7, 24] but the number of pack-years of smoking was not associated with chronic periodontitis in the present study. There are two possible reasons for this finding. First, current levels of smoking may not necessarily reflect past exposure in all individuals. Second, the number of pack-years of smoking may over-estimate lifetime exposure for current smokers who are not daily users. Betel quid chewing; a mixture of areca, slaked lime and tobacco wrapped in betel leaf is a common practice in South Asia. But the effect of this habit on chronic periodontitis has received limited attention. Consistent with the findings of Akhter et al. [25], betel quid chewing was associated with chronic periodontitis. However, Amarasena et al. [7] found that betel chewing was not associated with periodontitis in Sri Lankans. In addition to betel leaf, chewers use ingredients such as areca, lime and tobacco in the quid. It is possible that these ingredients may also have deleterious effects on the periodontium. Therefore further studies on the effects of these additives on periodontal health are warranted. Alcohol use was not associated with chronic periodontitis. In contrast a recent meta-analysis suggests that alcohol consumption is associated with an increased risk of periodontitis [26].

The effects of three co-morbid conditions were also assessed. Overweight/obesity and abdominal obesity were not associated with periodontitis. But systematic reviews suggest that overweight, obesity and increased waist circumference may be risk factors for development of periodontitis [11]. Consistent with other reports [27], self- reported diabetes emerged as a risk factor for chronic periodontitis. The relationship between diabetes and periodontal disease is considered to be bi-directional; hyperglycemia in diabetics can lead to increased inflammation thus contributing to increased periodontal destruction [28] and periodontal infection could adversely affect glycemic control in diabetics [29]. Self-reported hypertension was associated with chronic periodontitis and in agreement with previous studies [30]. Changes in microcirculation associated with hypertension may cause ischaemia in the periodontium favouring the development of periodontal disease [31].

## Conclusion

Several socio-demographic and behavioural factors as well as co-morbid conditions emerged as independent risk factors for chronic periodontits in this population. The findings of this study could be used for identifying high risk individuals, patient education and planning programmes to reduce the burden of chronic periodontits in Colombo district Sri Lanka.

## Limitations

This study has some limitations. The prevalence of diabetes and hypertension may have been under-estimated as they were based on self-reports. However detecting previously undiagnosed cases were beyond the scope of this study. It is possible that participants may have under-reported unhealthy habits such as smoking and alcohol use leading to information bias. Although steps were taken to minimize this bias to the best possible extent, it may not have been eliminated completely. As the study was confined to adults in Colombo district it may not be possible to directly generalize the findings to the wider Sri Lankan adult population.

## Abbreviations

CDC/AAP: Centers for Disease Control and Prevention/American Academy of Periodontology
CAL: clinical attachment loss
PPD: probing pocket depth
BMI: body mass index
